# Supplementary material for: Oxidative Modification, Structural Conformation, and Gel Properties of Pork Paste Protein Mediated by Oxygen Concentration in Modified Atmosphere Packaging
Source: Foods. 2024 Jan 25;13(3):391. doi: 10.3390/foods13030391 (PMC10855563; doi:10.3390/foods13030391)
Supplement: Supplementary file 1 [file foods-13-00391-s001.zip › foods-2801701-supplementary.pdf]

Table S1. The average particle size of pork paste gels from minced meat before storage (Day 0) and after refrigerated storage for 5 days in modified atmosphere with different O_2 concentrations.

Parameter	Day 0	0%	20%	40%	60%	80%	*P* value
$D_{3,2}$ (μm)	20.70 ± 0.06^{f}	26.03 ± 0.15^{e}	27.67 ± 0.23^{d}	29.77 ± 0.29^{c}	31.60 ± 0.29^{b}	48.67 ± 0.26^{a}	<0.001
$D_{4,3}$ (μm)	37.43 ± 0.41^{e}	53.47 ± 1.18^{d}	64.83 ± 2.37^{c}	81.00 ± 0.17^{b}	86.43 ± 2.20^{b}	123.33 ± 2.85^{a}	<0.001

Note: $D_{3,2}$ is the mean diameter in surface, $D_{4,3}$ is the mean diameter in volume. Mean values ± standard error. Different lowercase letters ($^{a-f}$) in the same line indicated significant differences between treatment groups at $p < 0.05$.
